# The Gambian Bone and Muscle Ageing Study: Baseline Data from a Prospective Observational African Sub-Saharan Study

**DOI:** 10.3389/fendo.2017.00219

**Published:** 2017-08-31

**Authors:** Ayse Zengin, Anthony J. Fulford, Yankuba Sawo, Landing M. Jarjou, Inez Schoenmakers, Gail Goldberg, Ann Prentice, Kate A. Ward

**Affiliations:** ^1^Nutrition and Bone Health Group, MRC Elsie Widdowson Laboratory, Cambridge, United Kingdom; ^2^Faculty of Medicine, Nursing and Health Sciences, Department of Medicine, School of Clinical Sciences at Monash Health, Monash University, Monash Medical Centre, Clayton, VIC, Australia; ^3^International Nutritional Group, London School of Hygiene and Tropical Medicine, London, United Kingdom; ^4^Calcium, Vitamin D and Bone Health Group at MRC Unit The Gambia, Banjul, Gambia; ^5^Faculty of Medicine and Health Sciences, Department of Medicine, University of East Anglia, Norwich, United Kingdom; ^6^MRC Lifecourse Epidemiology Unit, University of Southampton, Southampton, United Kingdom

**Keywords:** bone, ageing, Africa, muscle, dual energy X-ray absorptiometry, non-communicable disease, Gambia

## Abstract

The Gambian Bone and Muscle Ageing Study is a prospective observational study investigating bone and muscle ageing in men and women from a poor, subsistence farming community of The Gambia, West Africa. Musculoskeletal diseases, including osteoporosis and sarcopenia, form a major part of the current global non-communicable disease burden. By 2050, the vast majority of the world’s ageing population will live in low- and middle-income countries with an estimated two-fold rise in osteoporotic fracture. The study design was to characterise change in bone and muscle outcomes and to identify possible preventative strategies for fracture and sarcopenia in the increasing ageing population. Men and women aged ≥40 years from the Kiang West region of The Gambia were recruited with stratified sampling by sex and age. Baseline measurements were completed in 488 participants in 2012 who were randomly assigned to follow-up between 1.5 and 2 years later. Follow-up measurements were performed on 465 participants approximately 1.7 years after baseline measurements. The data set comprises a wide range of measurements on bone, muscle strength, anthropometry, biochemistry, and dietary intake. Questionnaires were used to obtain information on health, lifestyle, musculoskeletal pain, and reproductive status. Baseline cross-sectional data show preliminary evidence for bone mineral density and muscle loss with age. Men had greater negative differences in total body lean mass with age than women following adjustments for body size. From peripheral quantitative computed tomography scans, greater negative associations between bone outcomes and age at the radius and tibia were shown in women than in men. Ultimately, the findings from The Gambian Bone and Muscle Ageing Study will contribute to the understanding of musculoskeletal health in a transitioning population and better characterise fracture and sarcopenia incidence in The Gambia with an aim to the development of preventative strategies against both.

## Introduction

Health priorities in low and middle-income countries (LMICs) have until recently focused on infectious diseases. As these populations undergo social, economic, and environmental transition, non-communicable diseases (NCDs) of older age are becoming prevalent. As life expectancy is increasing, within 35 years the vast majority of the world’s older population will live in LMICs, with a consequent increase in associated NCDs (1, 2). The life expectancy in 2015 in The Gambia was 59.8 years in men and 62.5 years in women. In the Kiang West region, it was 65.3 and 73.5 years, respectively (3). In addition to musculoskeletal diseases, the prevalence of other diseases in adults in The Gambia include hypertension (27%), malaria (<2%), HIV, and AIDS (1.8%) (3–5). In the current study, musculoskeletal disease is a term used to describe osteoporosis (increased risk of fragility fracture) and sarcopenia (loss of muscle mass and strength), which are major contributors to the global NCD burden (6). For example, osteoporotic fracture is expected to double in LMICs by 2040 (7). The current cohort provides a baseline from which to characterise these changes as the population undergoes transition and to determine the individual, societal, and economic impact of osteoporosis and sarcopenia, and the associated increase in falls/fragility fractures, disability, morbidity, and mortality—all of which are currently unknown.

The first study to investigate bone health in The Gambia was over 30 years ago and used single-photon absorptiometry to measure the radius of women aged 18–85 years and showed that Gambians had a 5.6% lower bone mineral content (BMC) compared to Caucasian women (8); however, this difference was attenuated following adjustments for body size. A subsequent study in women aged ≥44 years utilised dual photon absorptiometry and showed that BMC at the lumbar spine was 24% lower in Gambians compared to Caucasians following adjustments for age, weight, and height (9). At that time, the incidence of fragility fractures was reported to be rare, paralleling the current low prevalence of osteoporosis throughout sub-Saharan Africa, though it is important to note the scarcity of available data (10, 11). The very limited data on bone health of Gambian women utilised imaging devices that were predecessors of the current clinical gold standard, dual energy X-ray absorptiometry (DXA) (8). In addition, there are no data in Gambian men. Currently, habitual calcium intakes are low, and parathyroid hormone levels are elevated while 25-hydroxyvitamin D status is good; low calcium and high PTH are risk factors for fracture in high-risk populations (12–15). The Gambia is undergoing a transition towards Western lifestyles particularly with respect to nutrition and physical inactivity, which are likely to contribute to the predicted rise in fracture risk.

Dual energy X-ray absorptiometry is used to measure areal bone mineral density (aBMD) as a predictor of fracture risk in older people from populations at high risk of osteoporosis (16). However, the use of DXA is of limited value in different ethnicities and at different times of life (13, 17). Newer imaging technologies such as peripheral quantitative computed tomography (pQCT) are valuable as they can measure volumetric BMD (vBMD), cortical, and trabecular compartments separately and provide information about the structural and geometrical parameters that contribute to bone strength (17). The application of DXA combined with pQCT in different ethnic populations is an area of growing research that allows better understanding of the underlying determinants of bone strength.

Understanding musculoskeletal health and better characterising fracture incidence in The Gambia may help in the development of preventative strategies against the predicted rise in osteoporotic fracture and sarcopenia. Ultimately, this would improve quality of life and reduce consequent morbidity and mortality associated with the conditions in the ageing population in The Gambia. Therefore, the Gambian Bone and Muscle Ageing Study (GamBAS) cohort was established to prospectively study musculoskeletal ageing in rural Gambian men and women. The primary aim of the study was to determine change in bone mineral density in men and women, the secondary aims were to characterise change in muscle, determine fracture and sarcopenia incidence, and finally to determine the biochemical and hormonal predictors of the detected changes in bone and muscle. This manuscript presents a general cohort description and findings from the baseline data.

## Study Outline

### Study Design

#### Recruitment

**Figure 1:**
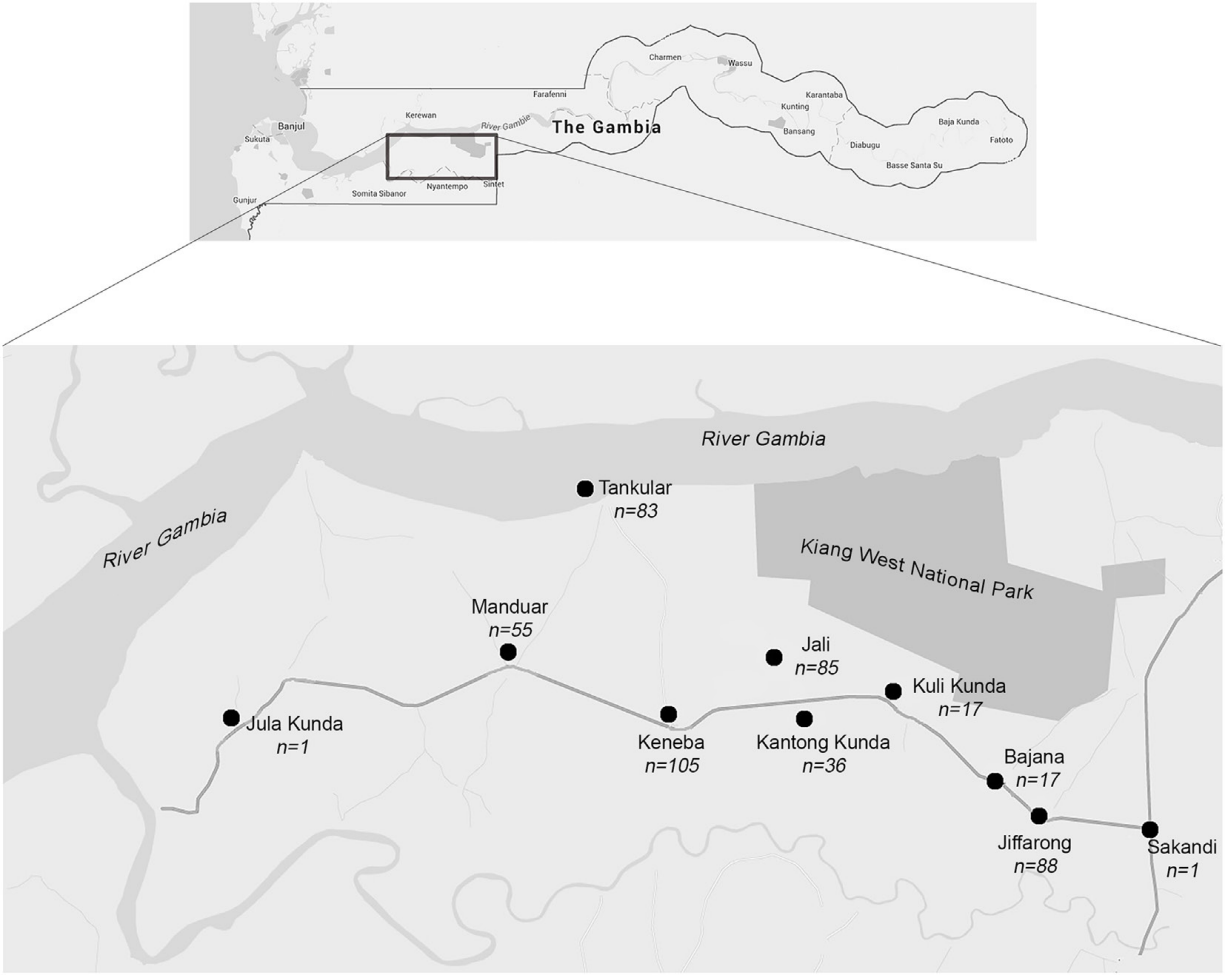
Map of the study area. The Kiang West region is located in the lower region of The Gambia.

In 1974, the Medical Research Council United Kingdom (MRC UK) established a permanent field station in Keneba (MRC Keneba), which is located in the middle of a 750 km^2^ district in the Lower River Region of Kiang West (Figure 1). Research facilities in Kiang West were initially set up to support nutrition studies in four core villages which have been part of a demographic surveillance system since 1950 and were the basis for the establishment of the comprehensive demographic surveillance, the Kiang West Demographic Surveillance System (KWDSS). The KWDSS and health care provision service has expanded into the wider Kiang West community (3). The population of Kiang West was described in 2015 (3), and residents are mainly of Mandinka ethnicity (79.9%) followed by Fula (16.2%), Jola (2.4%), and others (1.3%). The GamBAS cohort was recruited from 10 villages in Kiang West (Figure 1).

**Figure 2:**
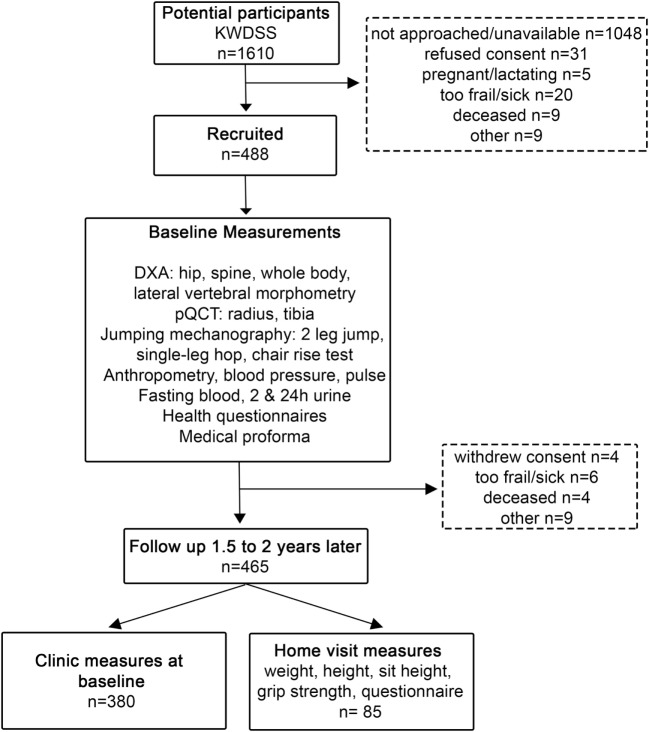
Flow diagram of participant recruitment.

All men and women aged 40 years and over and residing within the four core survey villages of Kiang West were identified using the KWDSS (Figure 2) (3). Potential participants were initially selected from the four core villages (Keneba, Manduar, Jali, and Kantong Kunda) to ensure a population representative of those who were known to have been born locally. Target group sizes were not reached; therefore, individuals from six other villages in the area were also recruited (Figure 1). Individuals were ranked according to the accuracy of date of birth ascertained from the KWDSS, from most to least accurate (that is, exact day, month or year), where recruitment priority was given to those with the most accurate. After initial village sensitisation and discussion with the elders, participants were located and approached by fieldworkers who explained the study in the local language and invited them to participate.

#### Eligibility Criteria

All eligible participants aged 40 years and over were given a verbal explanation of all the elements of the study and completed a screening questionnaire. If eligible, participants were given an information sheet and asked to sign or thumbprint an informed consent form and were enrolled in the study. Recruitment commenced in March 2011 and baseline measurements in 488 individuals were completed over 18 months (Figure 2).

#### Exclusion Criteria

Pregnant and lactating women were excluded. A woman was considered non-pregnant, non-lactating if she was at least 3 months post lactation and had regular menses. Individuals who were deemed too physically frail or incapable, due to existing disability or chronic illness to attend MRC Keneba for measurements were excluded from participating in the study (Figure 2). This was determined by the individual themselves, their spouse/relative or compound elder or a member of the fieldwork team. Prior to enrolment, participants were confirmed to not already be part of an on-going study at MRC Keneba (or elsewhere). The use of prescribed or self-medication to control pain (e.g., aspirin, paracetamol), indigestion (e.g., magnesium trisilicate, dried baobab fruit), chronic diseases of ageing (e.g., diuretics, local medicine), or use of chemical contraception (e.g., Depo Provera) was not an exclusion criterion.

#### Follow-up

The first follow-up measurements were scheduled 1.5–2 years later (1.7 ± 0.2 years) after the baseline measurement and were completed by the end of 2015 (Supplementary Material, Figure 2). Of the 488 participants who attended baseline visits, 380 had a repeated set of complete measurements taken. The remaining 108 participants who did not visit Keneba were approached for home visit: 85 were measured at home, 9 were away or could not be reached, 6 were excluded because they were too frail for the home visit, 4 died, and 4 withdrew consent (Figure 2), with a dropout rate of 4.7%. In the 85 participants that were measured at home, data collected were anthropometry, grip strength, and health questionnaires. Further follow-up measurements are being scheduled for 2017–2018.

### Measurements

#### Anthropometry and Questionnaires

Sitting and standing height (cm), weight (kg), mid upper arm circumference, and four skinfold measurements were measured (triceps, biceps, subscapular, and suprailiac). Blood pressure was measured using the OMRON 705IT blood pressure monitor during sitting, supine, and standing, and systolic and diastolic readings were recorded. A musculoskeletal and general health questionnaire collected data on: activities of daily living (e.g., working on a farm, gardening, performing five daily prayers, and fetching water), mental health, diagnosed illnesses (e.g., tuberculosis, diabetes mellitus, and leprosy), medication use, falls, fractures, musculoskeletal pain (located on specific body parts), lifestyle (occupational activities and domestic work), social demographics (including marital status, housing, and number of children), and medical history (through review of clinic medical records by the study physician/nurse). In women, there were also questions on menstrual cycle, breast-feeding history, pregnancy, parity, and menopausal status. Self-reported information on the use of medications and chronic medical conditions were collected.

#### Blood and Urine Collection

A 20 mL sample of venous blood was drawn from each participant in the early morning after an overnight fast; both lithium heparin and ethylene diamine tetra acetic acid (EDTA) plasma samples and an EDTA cell pellet for DNA extraction were stored at −80°C. Blood volume and all laboratory processing details were recorded on a data collection form. A 2-h fasting urine sample was collected, processed, and acidified and non-acidified samples were stored at −20°C. The 24-h urine samples were stored at −20°C in acidified and non-acidified aliquots; a further spun aliquot was stored at −80°C for assessment of microalbuminuria. To date, markers of calcium, vitamin D, bone metabolism, and kidney function have been measured.

#### Dietary Assessment

A 2-day prospective weighed dietary assessment was conducted in the participant’s home at a time close to the measurement day by trained and experienced fieldworkers. This method has been used in previous studies of children, adults, and older people in Kiang West (18, 19). Briefly, fieldworkers visited participant’s homes and recorded and weighed all food and drink items the participants consumed (total prepared minus amount left) over 48 h. Data were coded and analysed using Diets-In, Nutrients-Out programme with Gambian food tables (20, 21).

#### Dual-Energy X-ray Absorptiometry

**Figure 3:**
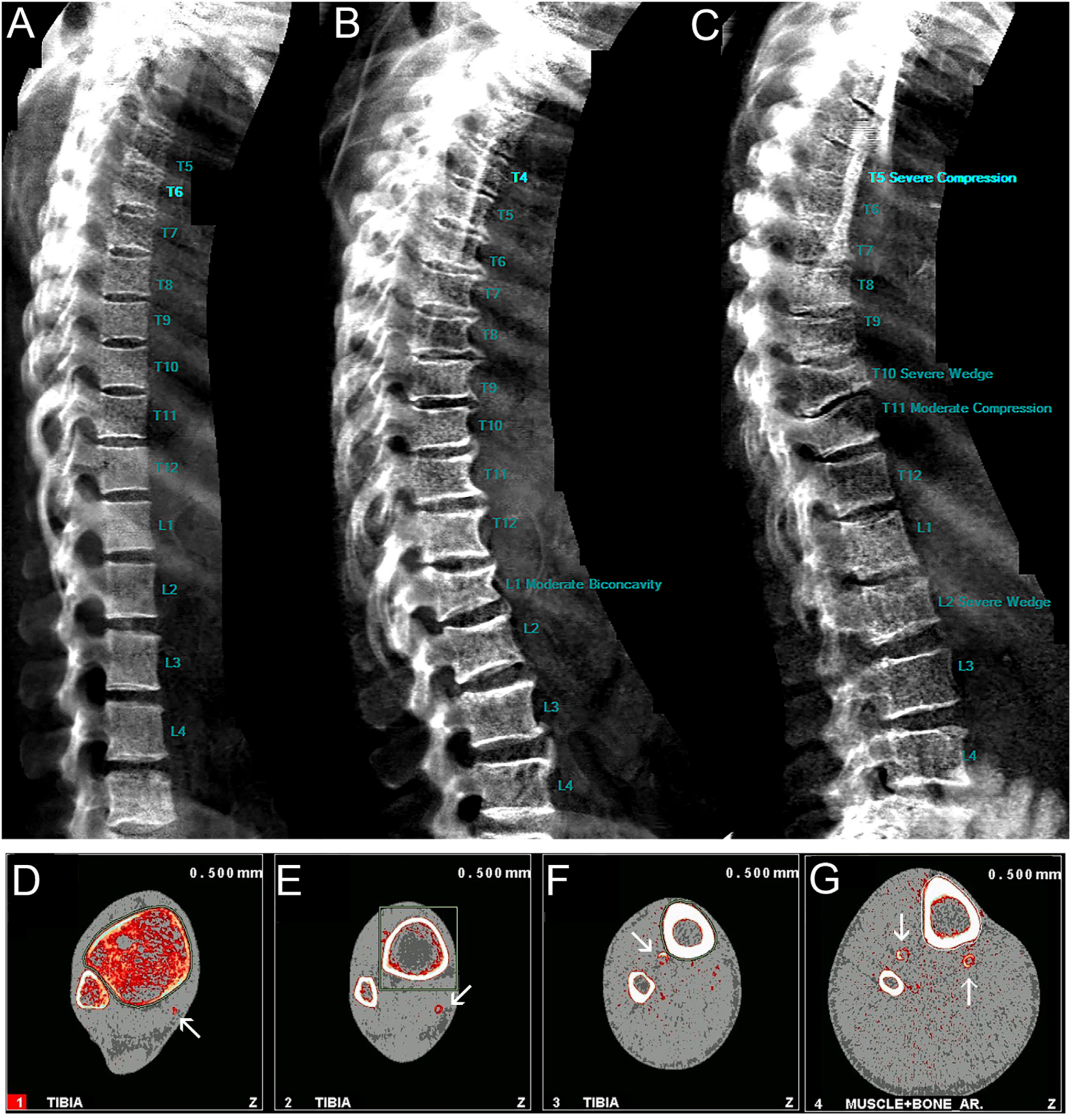
Representative lateral vertebral assessment scans of **(A)** normal vertebrae, **(B)** biconcavity at the spine, **(C)** severe compression and wedge fractures; representative peripheral quantitative computed tomography scans with *white* arrows indicating vascular calcification from the **(D)** 4%, **(E)** 14%, **(F)** 38%, and **(G)** 66% tibia.

Each participant was scanned at the whole body, hip, spine, and forearm by DXA as previously described (22) (GE Lunar Prodigy, Waltham, MA, USA) software version 10; iDXA (GE Lunar Prodigy, Waltham, MA, USA) was introduced during the first follow-up and cross calibration data were obtained in 119 adults and children at the follow-up measurement. Bone outcomes were as follows: areal BMD (aBMD, g/cm^2^), BMC (g), and bone area (BA, cm^2^). Body composition outcomes were as follows: total body fat mass (kg), total body lean mass (kg), and appendicular lean mass (kg). Fat and lean mass were also subdivided into the following regions: android, which is the lower abdominal area of the trunk between the ribs and the pelvis; gynoid, which is the region that encapsulates the hips, upper thighs, and buttocks. Lateral vertebral assessment (LVA) scans were obtained to determine whether there was any spinal degeneration, assessing osteoarthritic changes (osteophytes). Vertebral fractures were assessed semiautomatically in the GE Lunar software. All scans and fractures were assessed by a single reader, trained by an expert consultant musculoskeletal radiologist (Figures 3A–C). Figure 3A is a representative LVA scan showing no signs of spinal degeneration compared to Figures 3B,C. Short-term precision, measured as coefficient of variation (CV%) of duplicate measurements in 70 Gambian adults was <1% for all sites for BMD.

#### Peripheral Quantitative Computed Tomography

Peripheral quantitative computed tomography measurements were made at the radius and tibia using a Stratec XCT-2000 scanner (Stratec, Pforzheim, Germany) software version 6.20c, as previously described (23). Measurements were taken at the following sites: 4, 33, and 66% radius; 4, 14, 38, and 66% tibia. Forearm length was defined as the distance from the distal edge of the ulna styloid process to the olecranon. Leg length was defined as the distance from the most proximal edge of the medial malleolus to the intercondylar eminence. The scan sites were determined using a planar scout view of the distal radius or tibia and the reference line placed to bisect the lateral border of the end plate. The following outcome measures were taken at the 4% site: trabecular vBMD (Trab.vBMD, mg/cm^3^), total vBMD (Total vBMD, mg/cm^3^), cross-sectional area (CSA, mm^2^); 14, 33, 38, and 66% site: CSA (mm^2^), cortical vBMD (Ct.vBMD, mg/cm^3^), cortical BMC (Ct.BMC, mg/mm), Ct.Area (mm^2^), Ct.Thickness (mm), medullary area (mm^2^), stress strain index (SSI, mm^3^), cross-sectional moment of inertia (CSMI, mm^4^), periosteal circumference (mm), endosteal circumference (mm); and 66% site: cross-sectional muscle area (CSMA, mm^2^) and muscle density (mg/cm^3^). The range of CVs of duplicate measurements of bone outcomes in 62 Gambian adults was 1–4% for the radius and 1–3% for tibia measurements. The pQCT scans were also used to identify the presence or absence of vascular calcification at the 4, 14, 38, and 66% sites of the tibia (Figures 3D–G).

#### Jumping Mechanography

To assess lower limb muscle function, a Leonardo Ground Reaction Force Platform (Leonardo software version 4.2; Novotec Medical GmbH, Pforzheim, Germany) was used as previously described (24–26). Participants were asked to perform three tests: a two-leg countermovement jump, a hopping test, and a chair rise test. Each measurement assesses different aspects of muscle strength, maximum power (jump), maximum force (hop), and time-to-stand (chair-rise). If a participant did not feel comfortable/confident in performing any or all of the jumping tests, they were not excluded from participating in other measurements.

#### Grip Strength

Grip strength was measured using a dynamometer (Jamar Hand Dynamometer, IL, USA) (27). The individual was seated in an upright position with the arm supported on the armrest of the chair with the wrist in a neutral position and the thumb facing upwards. Participants were instructed to exert maximal force. For each individual, we allowed one practice and then took three test measurements. The outcome measured was force (kg).

### Ethics

Ethics was obtained from the MRC Unit The Gambia Scientific Co-ordinating Committee (SCC) and joint Gambian Government/MRC Unit The Gambia Ethics committee (SCC#1222). All participants provided written informed consent. All procedures were carried out in accordance with the Declaration of Helsinki (28). If any participant had potential health problems identified by fieldworkers during recruitment or by the team during the field visit, they were advised to visit the clinic at MRC Keneba for follow-up and were offered transport. Participants with elevated blood pressure [according to WHO guidelines (29)] were followed up and treated as appropriate. Any other abnormalities were discussed with the study physician who followed-up appropriately. For instance, if musculoskeletal abnormalities were detected on the scan, the participant was referred for radiography at MRC Fajara.

### Power Calculation and Analyses

Power calculations for GamBAS were determined to detect within individual change in femoral neck aBMD as it has the worst precision of all the sites measured with DXA. A sample size of 66 would be needed to detect a 1% change per annum over a random follow-up interval between 1.5 and 2 years later with a precision of 30% in the expected rate of change (described fully in Supplementary Material). To detect a 2% change over the same time with 30% precision, we would need a sample size of 16, or 37 for a precision of 20% in the expected rate of change. Thus, a minimum of 30 participants per 5-year age band will be sufficient to identify rates of change within an individual of 1–2% per annum at the hip with confidence. In the other DXA and pQCT regions, which can be measured with more precision, smaller rates of change will be detectable with this number of participants. These rates are similar to or less than those observed in Caucasian populations during ageing and are biologically plausible and clinically relevant in terms of fracture risk (30, 31). Stratified sampling was used to ensure equal distribution of participants across each of the 5-year age bands, namely: 40–44.99, 45–49.99, 50–54.99, 55–59.99, 60–64.99, 65–69.99, 70–74.99, 75 years and over; 239 men and 249 women were recruited. The scheduled follow-up measurements at a randomised interval between 1.5 and 2 years later for each participant will minimise the likelihood that the two measurements take place at exactly the same time of year. The exact interval for follow-up was different for each individual and assigned randomly at recruitment. This design was chosen since it allows us to account for inter-individual variation in patterns of seasonal change (Supplementary Material). Preliminary analyses on the baseline data include linear regression models to determine relationships between height, weight, musculoskeletal outcomes, and age. Analyses were split by sex due to patterns of age-related change in bone and muscle varying by sex. The data presented are from the baseline visits so all participants had measurements recorded for the primary and secondary outcomes. Some DXA and pQCT scans were excluded where significant motion artefact was detected which resulted in some missing data and were considered missing in these analyses. The missing data were independent of the participant’s age and did not affect the preliminary analyses but may have reduced statistical power in some age bands. The participants who had missing data were not excluded from other measurements.

## Results: Key Findings from Baseline Measurements

### Population Characteristics

Kiang West residents are mainly Muslim (3); GamBAS participants reported that they pray five times a day and most men and women were able to perform these prayers without physical difficulty (Table 1). Praying five times a day involves standing, kneeling, and rising from the floor multiple times. The questionnaire regarding musculoskeletal pain revealed that a higher proportion of women experienced musculoskeletal pain compared to men at the: back (51 vs 39%), hip (49 vs 39%), and knees (51 vs 40%); this may potentially be due to the subsistence fieldwork being undertaken primarily by women (Table 1). The villages are divided into compounds where extended multigenerational families live together. Polygamy is practiced in this population; women have one husband at any one time whereas men may have up to four wives. Women often live in one hut with their co-wives and men often live separately; 99.5% of men and 98.8% of women reside in dwellings with corrugate roofs. Women had a median of 9 (IQR: 7–10) children that were born alive. The main livelihood is rural subsistence farming where income and eating patterns depend on available foods that fluctuate throughout the year and are greatly influenced by the annual rainy season (June to October). This influences intakes of specific foods, nutrients, and energy intake. Dietary intakes of key micronutrients remain low and relatively unchanged to our previous work. Overall, men had higher intakes of all micronutrients. Some notable sex differences include a 21% greater daily habitual calcium intake in men than in women (Table 2). The greatest sex difference was seen in daily habitual iron intake, where men had a 33% greater daily iron intake compared to women. Across the age bands, daily habitual calcium intake [mean (SD)] was 378.0 (176.0) mg/day in men and 295.9 (175.9) mg/day in women (Table 2).

**Table 1:** Lifestyle outcomes in men and women.

	**Men** (*n* = 239)	**Women** (*n* = 249)

**Number of marriages**		
Never married	6 (3)	8 (3)
Once	217 (91)	120 (48)
Twice	8 (3)	65 (26)
Three or more	2 (1)	56 (22)
Missing data	6 (3)	–
**Living arrangement**		
Alone	5 (2)	16 (6)
With 1 spouse and no children	4 (2)	3 (1)
With 1 spouse and children	128 (54)	–
With co-wives/spouses	3 (1)	134 (54)
With children only	2 (1)	83 (33)
With co-wives/spouses and children	89 (37)	–
Others	–	12 (5)
Missing data	8 (3)	1 (0)
**Smoking status**		
Non-smoker	181 (76)	202 (81)
Current smoker	55 (23)	39 (16)
Missing data	3 (1)	8 (3)
**Farm or field work**		
No	36 (15)	25 (10)
Used to but not anymore	8 (3)	7 (3)
Yes currently	192 (80)	211 (85)
Missing data	3 (1)	6 (2)
**Perform five daily prayers**		
Yes, with difficulty but without help	15 (6)	8 (3)
Yes, without difficulty	220 (92)	232 (93)
Only with help	–	1 (0)
Missing data	4 (2)	8 (3)
**Musculoskeletal pain**		
Back pain	94 (39)	128 (51)
Hip pain	92 (39)	121 (49)
Knee pain	95 (40)	126 (51)

*Values are frequency (%)*.

**Table 2:** Nutritional intake of men and women.

	Men (*n* = 225)^a^	Women (*n* = 242)^a^	*p*-value
Calcium (mg/day)	378.0 ± 176.0	295.9 ± 175.9	**<0.0001**
Phosphorus (mg/day)	836.4 ± 275.4	620.2 ± 243.4	**<0.0001**
Iron (mg/day)	37.2 ± 25.8	25.0 ± 16.5	**<0.0001**
Zinc (mg/day)	9.3 ± 3.0	7.0 ± 2.8	**<0.0001**
Dietary fibres (mg/day)	44.4 ± 14.2	33.9 ± 12.4	**<0.0001**
Phytate (g/day)	1.3 ± 0.5	1.0 ± 0.4	**<0.0001**
Potassium (mg/day)	2,409.0 ± 868.9	1,800.1 ± 705.4	**<0.0001**
Magnesium (mg/day)	527.3 ± 192.9	388.4 ± 150.4	**<0.0001**

*Values are mean ± SD*.

*Bold indicates significance*.

*Dietary intakes were estimated from 2-day weighed diet diaries, and intakes calculated from Gambian food tables*.

*^a^21 participants did not have dietary information available*.

### Bone, Body Composition, and Age

Baseline data describing anthropometry and body composition in men and women are shown in Tables 3 and 4. In men, the most significant negative associations with age were for weight (−0.29 kg, *p* < 0.0001) and total body lean mass (−0.24, *p* < 0.0001). In women, anthropometry and body composition outcomes all had significant negative associations with age, with the greatest difference observed in weight (−0.26 kg, *p* < 0.0001). Men had greater negative differences in total body lean mass with age than women following adjustments for weight and height (*R*^2^ = 0.90, interaction *p* < 0.0001). Waist circumference and body fat outcomes were not different with age in both men and women.

**Table 3:** Anthropometry and body composition in men.

**Age (yr)**	**40–44** (*n* = 25)	**45–49** (*n* = 34)	**50–54** (*n* = 29)	**55–59** (*n* = 31)	**60–64** (*n* = 27)	**65–69** (*n* = 35)	**70–74** (*n* = 30)	**75+** (*n* = 28)	**β-Coefficient (95% CI)**	***p*-value**

Weight (kg)	66.9 ± 10.8	61.5 ± 9.5	64.1 ± 12.3	59.1 ± 10.8	59.2 ± 8.4	59.5 ± 9.6	55.8 ± 7.6	53.8 ± 7.5	−0.29 (−0.39, −0.19)	**<0.0001**
Height (cm)	171.2 ± 5.1	171.0 ± 5.9	171.2 ± 6.7	170.3 ± 6.8	169.1 ± 8.7	167.4 ± 7.0	168.7 ± 7.9	164.9 ± 6.0	−0.16 (−0.23, −0.08)	**<0.0001**
Sitting height (cm)	86.2 ± 3.7	85.2 ± 3.1	85.1 ± 4.1	84.0 ± 3.4	83.7 ± 4.0	82.9 ± 3.6	83.1 ± 4.2	81.2 ± 2.7	−0.11 (−0.15, −0.08)	**<0.0001**
Sit:stand height ratio	0.50 ± 0.02	0.50 ± 0.01	0.50 ± 0.01	0.49 ± 0.01	0.50 ± 0.01	0.50 ± 0.01	0.49 ± 0.01	0.49 ± 0.02	−0.0002 (−0.0004, −0.00008)	**0.003**
BMI	22.8 ± 3.7	21.0 ± 3.0	21.8 ± 3.5	20.3 ± 2.9	20.7 ± 2.8	21.2 ± 3.2	19.6 ± 2.3	19.8 ± 2.5	−0.06 (−0.09, −0.03)	**<0.0001**
Waist circumference (cm)	79.2 ± 8.5	76.5 ± 8.0^(*n*=32)^	80.1 ± 8.4	77.8 ± 8.5	77.1 ± 17.1	79.2 ± 9.3	76.1 ± 7.9^(*n*=29)^	75.8 ± 8.6	−0.06 (−0.16, 0.04)	0.243
Total body fat mass (kg)	11.0 ± 6.6^(*n*=24)^	7.2 ± 5.3	9.7 ± 7.5	7.2 ± 6.0	8.1 ± 5.4	9.3 ± 6.7	7.3 ± 4.6^(*n*=29)^	7.1 ± 4.2^(*n*=26)^	−0.05 (−0.12, 0.01)	0.120
Total% fat	15.5 ± 7.3^(*n*=24)^	11.0 ± 6.4	14.1 ± 8.4	11.1 ± 6.9	13.0 ± 7.3	14.6 ± 8.1	12.6 ± 6.6^(*n*=29)^	12.7 ± 6.2^(*n*=26)^	−0.007 (−0.08, 0.08)	0.865
Android fat mass (kg)	0.9 ± 0.7	0.6 ± 0.5	0.8 ± 0.7	0.6 ± 0.6	0.7 ± 0.6	0.8 ± 0.7	0.5 ± 0.4	0.6 ± 0.5^(*n*=26)^	−0.004 (−0.01, 0.002)	0.203
Gynoid fat mass (kg)	2.2 ± 1.1^(*n*=24)^	1.5 ± 1.0	1.9 ± 1.3	1.4 ± 1.0	1.6 ± 0.9	1.8 ± 1.0	1.5 ± 0.9^(*n*=29)^	1.4 ± 0.7^(*n*=27)^	−0.01 (−0.02, 0.0006)	0.051
FMI (kg/m^2^)	3.8 ± 2.3^(*n*=24)^	2.5 ± 1.7	3.3 ± 2.5	2.4 ± 1.9	2.8 ± 2.0	3.3 ± 2.4	2.6 ± 1.6^(*n*=29)^	2.6 ± 1.6^(*n*=26)^	−0.01 (−0.03, 0.01)	0.292
Total body lean mass (kg)	53.2 ± 5.6^(*n*=24)^	51.7 ± 5.8	51.5 ± 6.9	49.5 ± 5.9	48.4 ± 5.1	47.6 ± 4.9	45.8 ± 5.5^(*n*=29)^	44.1 ± 5.0^(*n*=26)^	−0.24 (−0.29, −0.18)	**<0.0001**
aLM (kg)	25.6 ± 3.1	24.6 ± 3.5	24.6 ± 3.9	23.2 ± 3.2	22.4 ± 2.6	21.9 ± 2.8	20.9 ± 3.0	20.0 ± 2.9^(*n*=27)^	−0.1 (−0.2, −0.1)	**<0.0001**
Android lean mass (kg)	3.4 ± 0.5	3.3 ± 0.4	3.3 ± 0.5	3.2 ± 0.4	3.3 ± 0.4	3.2 ± 0.4	3.2 ± 0.5	2.9 ± 0.4^(*n*=26)^	−0.009 (−0.01, −0.004)	**<0.0001**
Gynoid lean mass (kg)	7.7 ± 1.1^(*n*=24)^	7.4 ± 1.1	7.3 ± 1.2	6.9 ± 0.9	6.8 ± 0.9	6.7 ± 0.8	6.5 ± 0.8^(*n*=29)^	6.2 ± 0.7^(*n*=27)^	−0.04 (−0.05, −0.03)	**<0.0001**
aLMI (kg/m^2^)	8.7 ± 0.9	8.4 ± 1.1	8.4 ± 1.0	8.0 ± 0.8	7.8 ± 0.7	7.8 ± 0.8	7.3 ± 0.8	7.3 ± 0.8^(*n*=27)^	−0.04 (−0.05, −0.03)	**<0.0001**

*Values are mean ± SD*.

*β-Coefficients are calculated with age as a continuous variable*.

*Superscript values indicate the group numbers*.

*Bold indicates significance*.

*BMI, body mass index; FMI, fat mass index, calculated as whole body fat mass divided by height squared; aLM, appendicular lean mass; aLMI, appendicular lean mass index, calculated as appendicular lean mass divided by height squared*.

**Table 4:** Anthropometry and body composition in women.

	40–44 (*n* = 28)	45–49 (*n* = 32)	50–54 (*n* = 30)	55–59 (*n* = 31)	60–64 (*n* = 31)	65–69 (*n* = 33)	70–74 (*n* = 30)	75+ (*n* = 34)	β-Coefficient (95% CI)	*p*-value
Weight (kg)	58.1 ± 11.5	60.8 ± 11.4	57.1 ± 10.8	53.8 ± 9.6	53.4 ± 7.2	53.5 ± 9.6	52.2 ± 9.9	49.3 ± 8.5	−0.26 (−0.35, −0.16)	**<0.0001**
Height (cm)	159.3 ± 5.1	159.8 ± 6.1	158.6 ± 6.2	158.1 ± 5.8	157.6 ± 4.9	160.1 ± 5.7	154.8 ± 5.7	154.0 ± 5.7	−0.14 (−0.20, −0.09)	**<0.0001**
Sitting height (cm)	81.7 ± 2.8	81.2 ± 3.5	80.4 ± 2.9	79.1 ± 3.8	79.5 ± 3.1	80.2 ± 3.5	77.8 ± 3.3	76.5 ± 3.3	−0.13 (−0.16, −0.09)	**<0.0001**
Sit:stand height ratio	0.51 ± 0.02	0.51 ± 0.01	0.51 ± 0.02	0.50 ± 0.02	0.50 ± 0.01	0.50 ± 0.02	0.50 ± 0.02	0.50 ± 0.01	−0.0004 (−0.0005, −0.0002)	**<0.0001**
BMI	22.9 ± 4.4	23.9 ± 4.4	22.7 ± 4.3	21.4 ± 3.1	21.4 ± 2.3	20.8 ± 3.2	21.7 ± 3.7	20.7 ± 2.8	−0.07 (−0.10, −0.03)	**<0.0001**
Waist circumference (cm)	70.7 ± 10.1	75.7 ± 9.7	72.0 ± 8.6	70.6 ± 6.6	71.4 ± 6.3^(^*^n^*^=29)^	71.0 ± 7.1^(^*^n^*^=29)^	73.3 ± 8.5^(^*^n^*^=23)^	68.4 ± 5.4^(^*^n^*^=19)^	−0.06 (−0.14, 0.03)	0.203
Total body fat mass (kg)	18.4 ± 8.7^(^*^n^*^=27)^	20.7 ± 9.3	18.3 ± 8.3	16.3 ± 6.7^(^*^n^*^=30)^	16.0 ± 4.8	16.1 ± 6.8	16.4 ± 6.7^(^*^n^*^=29)^	14.1 ± 5.5^(^*^n^*^=30)^	−0.12 (−0.20, −0.05)	**0.001**
Total% fat	30.3 ± 8.1^(^*^n^*^=27)^	32.5 ± 10.0	30.8 ± 8.7	29.3 ± 8.2^(^*^n^*^=30)^	29.6 ± 6.0	29.0 ± 7.6	30.4 ± 7.9^(^*^n^*^=29)^	27.9 ± 7.1^(^*^n^*^=30)^	−0.07 (−0.15, 0.01)	0.09
Android fat mass (kg)	1.1 ± 0.9^(^*^n^*^=27)^	1.3 ± 0.8	1.2 ± 0.8	0.9 ± 0.5^(^*^n^*^=30)^	0.9 ± 0.4	1.0 ± 0.6	1.0 ± 0.6^(^*^n^*^=29)^	0.8 ± 0.5^(^*^n^*^=31)^	−0.008 (−0.01, −0.001)	**0.02**
Gynoid fat mass (kg)	4.1 ± 1.5^(^*^n^*^=27)^	4.3 ± 1.6	3.9 ± 1.3	3.4 ± 1.2	3.5 ± 1.0	3.4 ± 1.2	3.2 ± 1.1	2.9 ± 1.0^(^*^n^*^=33)^	−0.03 (−0.04, −0.02)	**<0.0001**
FMI (kg/m^2^)	7.2 ± 3.4^(^*^n^*^=27)^	8.1 ± 3.7	7.3 ± 3.4	6.5 ± 2.6^(^*^n^*^=30)^	6.4 ± 1.8	6.2 ± 2.5	6.9 ± 2.7^(^*^n^*^=29)^	6.0 ± 2.2^(^*^n^*^=30)^	−0.04 (−0.07, −0.01)	**0.009**
Total body lean mass (kg)	36.7 ± 4.1^(^*^n^*^=27)^	37.0 ± 4.4	35.7 ± 4.0	35.0 ± 4.5^(^*^n^*^=30)^	34.7 ± 3.6	34.7 ± 3.4	33.4 ± 4.7^(^*^n^*^=29)^	32.5 ± 4.3^(^*^n^*^=30)^	−0.11 (−0.16, −0.07)	**<0.0001**
aLM (kg)	16.9 ± 2.3^(^*^n^*^=27)^	16.9 ± 2.2	16.1 ± 2.3	15.6 ± 2.3	15.4 ± 2.1	15.3 ± 2.0	14.7 ± 2.4	14.1 ± 2.2	−0.07 (−0.10, −0.05)	**<0.0001**
Android lean mass (kg)	2.3 ± 0.3^(^*^n^*^=27)^	2.4 ± 0.4	2.3 ± 0.3	2.2 ± 0.3^(^*^n^*^=30)^	2.2 ± 0.2	2.2 ± 0.2	2.2 ± 0.4^(^*^n^*^=29)^	2.2 ± 0.3^(^*^n^*^=31)^	−0.006 (−0.009, −0.003)	**0.001**
Gynoid lean mass (kg)	5.2 ± 0.9^(^*^n^*^=27)^	5.2 ± 0.7	5.0 ± 0.6	4.7 ± 0.8	4.8 ± 0.7	4.8 ± 0.7	4.5 ± 0.7	4.4 ± 0.6^(^*^n^*^=33)^	−0.02 (−0.03, −0.01)	**<0.0001**
aLMI (kg/m^2^)	6.6 ± 0.8^(^*^n^*^=27)^	6.6 ± 0.8	6.4 ± 0.7	6.2 ± 0.6	6.2 ± 0.7	5.9 ± 0.6	6.1 ± 0.8	5.9 ± 0.7	−0.02 (−0.03, −0.01)	**<0.0001**

*Values are mean ± SD*.

*β-Coefficients are calculated with age as a continuous variable*.

*Superscript values indicate the group numbers*.

*Bold indicates significance*.

*BMI, body mass index; FMI, fat mass index, calculated as whole body fat mass divided by height squared; aLM, appendicular lean mass; aLMI, appendicular lean mass index, calculated as appendicular lean mass divided by height squared*.

**Figure 4:**
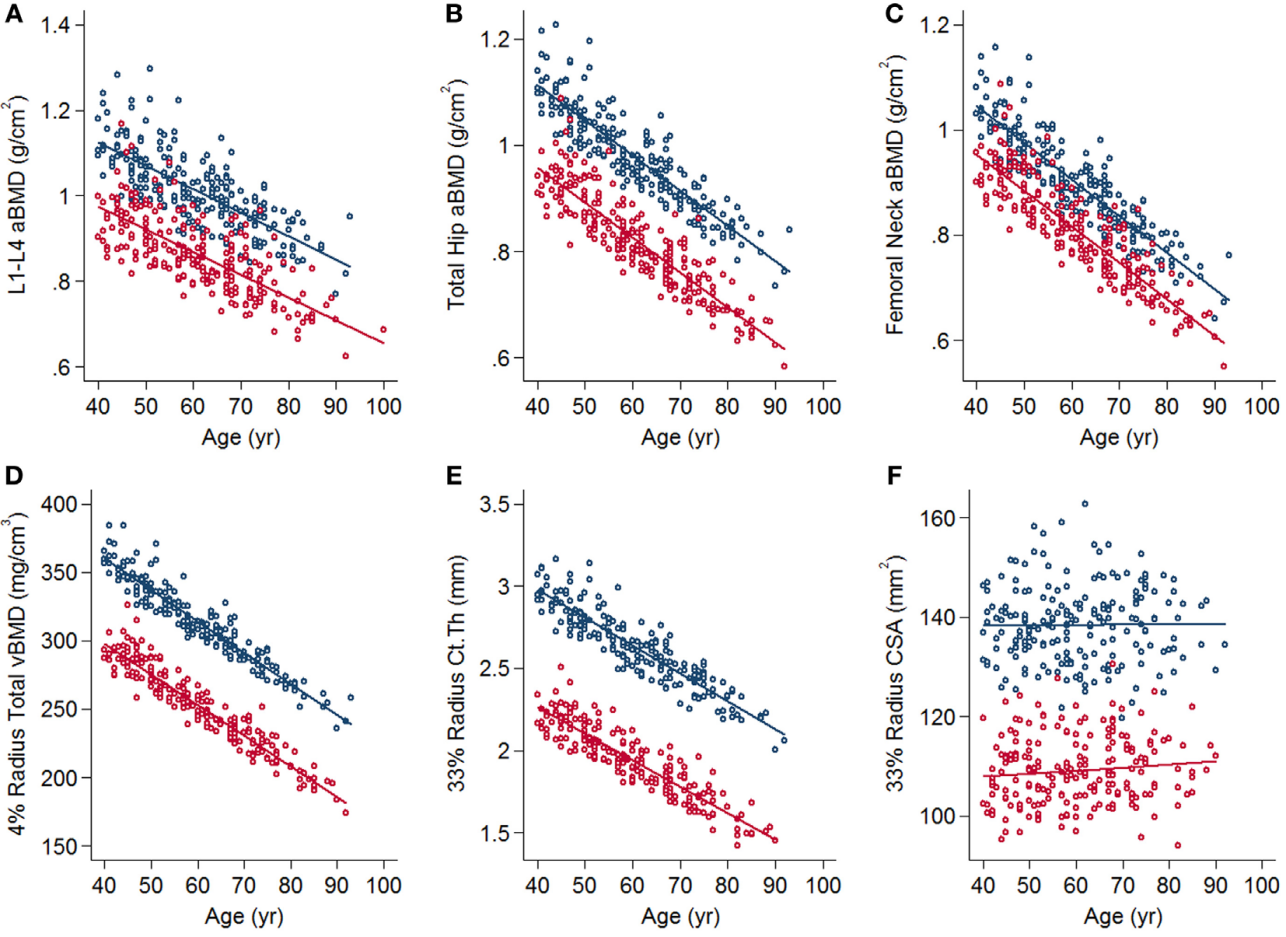
Associations between age and clinically relevant bone outcomes **(A)** L1–L4 areal bone mineral density (aBMD), **(B)** total hip aBMD, **(C)** femoral neck aBMD, **(D)** 4% radius total volumetric BMD, **(E)** 33% radius cortical thickness, and **(F)** 33% radius cross-sectional area. Scatter plots are from linear regression with adjustments for sex, weight, and height. Blue lines and dots represent men and red lines and dots represent women.

Dual energy X-ray absorptiometry bone parameters in men and women are displayed in Tables 5 and 6, respectively. Baseline cross-sectional data show preliminary evidence for bone and muscle loss with age. In both men and women, there were negative associations between age, and DXA bone outcomes at all sites, particularly clinically relevant sites including spine, total hip, and femoral neck aBMD (Figures 4A–C). There were positive associations with BA and age at the total hip and radius in both men and women following adjustments (Tables 5 and 6). Data from pQCT in men and women are shown in Tables 7 and 8, respectively. At the distal radius, there were greater negative differences with age in women compared to men in the 4% total vBMD. At the 33% cortical site, there was a greater reduction in cortical thickness in women than in men and a concurrent greater decrease in cortical vBMD. There were small, but greater increases in CSA in women (Figures 4D–F). At the 4% tibia, there were greater negative differences in Trab.vBMD with age in women than in men following adjustments for body size (*R*^2^ = 0.41, interaction *p* < 0.0001).

**Table 5:** Dual energy X-ray absorptiometry bone parameters in men.

**Age (yr)**	**40–44** (*n* = 25)	**45–49** (*n* = 34)	**50–54** (*n* = 29)	**55–59** (*n* = 31)	**60–64** (*n* = 27)	**65–69** (*n* = 35)	**70–74** (*n* = 30)	**75+** (*n* = 28)	**β-Coefficient (95% CI)**	***R*^2^**	**Unadjusted *p*-value**	**β-Coefficient (95% CI)**	***R*^2^**	**Adjusted *p*-value**

**Whole body**														
aBMD (g/cm^2^)	1.2 ± 0.1^(*n*=24)^	1.2 ± 0.1	1.2 ± 0.1	1.1 ± 0.1	1.1 ± 0.1	1.1 ± 0.1	1.1 ± 0.1^(*n*=29)^	1.1 ± 0.1^(*n*=26)^	−0.003 (−0.004, −0.002)	0.13	**<0.0001**	−0.002 (−0.003, −0.0008)	0.34	**<0.0001**
BMC (g)	2,804 ± 433^(*n*=24)^	2,632 ± 358	2,739 ± 433	2,532 ± 374	2,442 ± 448	2,433 ± 366	2,335 ± 299^(*n*=29)^	2,279 ± 380^(*n*=26)^	−13.3 (−17.4, −9.2)	0.15	**<0.0001**	−3.9 (−6.6, −1.1)	0.66	**0.007**
BA (cm^2^)	2,355 ± 200^(*n*=24)^	2,275 ± 212	2,321 ± 233	2,242 ± 217	2,210 ± 243	2,175 ± 215	2,171 ± 204^(*n*=29)^	2,087 ± 205^(*n*=26)^	−6.2 (−8.5, −3.9)	0.11	**<0.0001**	−0.1 (−1.2, 1.0)	0.83	0.856
**Spine**														
aBMD (g/cm^2^)	1.1 ± 0.2^(*n*=24)^	1.0 ± 0.1	1.1 ± 0.1	1.0 ± 0.1^(*n*=30)^	1.0 ± 0.2	1.0 ± 0.2	1.0 ± 0.2	1.0 ± 0.2^(*n*=26)^	−0.002 (−0.004, −0.0004)	0.03	**0.014**	−0.0006 (−0.002, 0.001)	0.13	0.486
BMC (g)	61.1 ± 12.6^(*n*=24)^	57.1 ± 8.9	59.6 ± 11.0	54.5 ± 9.3^(*n*=30)^	55.9 ± 17.6	55.4 ± 11.3	54.2 ± 10.8	53.5 ± 13.5^(*n*=26)^	−0.1 (−0.3, −0.02)	0.02	**0.025**	0.06 (−0.05, 0.17)	0.31	0.306
BA (cm^2^)	55.7 ± 5.2^(*n*=24)^	55.7 ± 4.0	56.4 ± 5.8	54.8 ± 5.2^(*n*=30)^	55.9 ± 7.2	54.9 ± 4.8	55.6 ± 6.8	54.6 ± 5.6^(*n*=26)^	−0.02 (−0.08, 0.03)	0.003	0.405	0.09 (0.05, 0.13)	0.56	**<0.0001**
**Total hip**														
aBMD (g/cm^2^)	1.1 ± 0.2	1.0 ± 0.1	1.1 ± 0.1	1.0 ± 0.1^(*n*=29)^	0.9 ± 0.2	1.0 ± 0.1^(*n*=33)^	0.9 ± 0.1^(*n*=29)^	0.9 ± 0.2^(*n*=27)^	−0.004 (−0.006, −0.003)	0.14	**<0.0001**	−0.003 (−0.005, −0.002)	0.20	**<0.0001**
BMC (g)	35.4 ± 6.2	34.2 ± 4.2	35.8 ± 4.5	33.0 ± 4.4^(*n*=29)^	31.4 ± 5.9	31.6 ± 4.7^(*n*=33)^	31.1 ± 4.4^(*n*=29)^	29.7 ± 6.3^(*n*=27)^	−0.15 (−0.2, −0.1)	0.12	**<0.0001**	−0.07 (−0.1, −0.02)	0.32	**0.005**
BA (cm^2^)	33.3 ± 2.1	33.5 ± 2.6	34.1 ± 2.3	33.4 ± 2.2^(*n*=29)^	33.8 ± 2.6	33.0 ± 2.1^(*n*=33)^	34.0 ± 2.0^(*n*=29)^	33. 0 ± 2.2^(*n*=27)^	−0.009 (−0.03, 0.02)	0.002	0.468	0.04 (0.02, 0.06)	0.52	**<0.0001**
**Femoral neck**														
aBMD (g/cm^2^)	1.0 ± 0.1	1.0 ± 0.1	1.0 ± 0.1	0.9 ± 0.1^(*n*=30)^	0.9 ± 0.1	0.9 ± 0.1	0.8 ± 0.1^(*n*=29)^	0.8 ± 0.2^(*n*=27)^	−0.005 (−0.007, −0.004)	0.22	**<0.0001**	−0.004 (−0.006, −0.003)	0.31	**<0.0001**
BMC (g)	5.0 ± 1.0	4.7 ± 0.7	5.0 ± 0.7	4.4 ± 0.6^(*n*=30)^	4.3 ± 0.8	4.3 ± 0.7	4.0 ± 0.6^(*n*=29)^	3.8 ± 0.8^(*n*=27)^	−0.03 (−0.04, −0.02)	0.20	**<0.0001**	−0.02 (−0.03, −0.01)	0.36	**<0.0001**
BA (cm^2^)	4.8 ± 0.6	5.0 ± 0.6	5.1 ± 0.5	4.8 ± 0.5^(*n*=30)^	5.0 ± 0.5	5.0 ± 0.5	4.8 ± 0.4^(*n*=29)^	4.8 ± 0.6^(*n*=27)^	−0.003 (−0.009, 0.002)	0.01	0.264	0.002 (−0.003, 0.008)	0.13	0.382
**Radius**														
aBMD (g/cm^2^)	0.7 ± 0.1	0.7 ± 0.1	0.7 ± 0.1	0.7 ± 0.1	0.7 ± 0.1	0.7 ± 0.1	0.6 ± 0.1	0.6 ± 0.1^(*n*=27)^	−0.004 (−0.005, −0.003)	0.25	**<0.0001**	−0.003 (−0.004, −0.002)	0.34	**<0.0001**
BMC (g)	12.9 ± 1.6	12.2 ± 1.7	12.5 ± 1.9	12.0 ± 1.8	11.4 ± 1.8	11.3 ± 2.1	10.6 ± 1.5	10.2 ± 2.0^(*n*=27)^	−0.07 (−0.09, −0.05)	0.17	**<0.0001**	−0.03 (−0.05, −0.02)	0.49	**<0.0001**
BA (cm^2^)	17.3 ± 1.6	17.1 ± 2.1	17.5 ± 2.1	17.4 ± 1.8	17.6 ± 2.0	17.3 ± 2.1	17.4 ± 2.0	16.8 ± 1.7^(*n*=27)^	−0.005 (−0.02, 0.02)	0.001	0.649	0.03 (0.01, 0.05)	0.41	**0.001**

*Values are mean ± SD*.

*Bold indicates significance*.

*Adjustments were made for weight and height*.

*β-Coefficients are calculated with age as a continuous variable*.

*Superscript values indicate the group numbers*.

*aBMD, areal bone mineral density; BMC, bone mineral content; BA, bone area*.

**Table 6:** Dual energy X-ray absorptiometry bone parameters in women.

**Age (yr)**	**40–44** (*n* = 28)	**45–49** (*n* = 32)	**50–54** (*n* = 30)	**55–59** (*n* = 31)	**60–64** (*n* = 31)	**65–69** (*n* = 33)	**70–74** (*n* = 30)	**75+** (*n* = 34)	**β-Coefficient (95% CI)**	***R*^2^**	**Unadjusted *p*-value**	**β-Coefficient (95% CI)**	***R*^2^**	**Adjusted *p*-value**

**Whole body**														
aBMD (g/cm^2^)	1.1 ± 0.1^(*n*=27)^	1.1 ± 0.1	1.0 ± 0.1	1.0 ± 0.1^(*n*=30)^	1.0 ± 0.1	0.9 ± 0.1	0.9 ± 0.1^(*n*=29)^	0.9 ± 0.1^(*n*=30)^	−0.005 (−0.006, −0.005)	0.37	**<0.0001**	−0.004 (−0.005, −0.003)	0.54	**<0.0001**
BMC (g)	2,273 ± 361^(*n*=27)^	2,210 ± 366	2,081 ± 449	1,827 ± 300^(*n*=30)^	1,752 ± 252	1,749 ± 369	1,679 ± 368^(*n*=29)^	1,538 ± 303^(*n*=30)^	−18.9 (−22.5, −15.3)	0.31	**<0.0001**	−10.6 (−12.9, −8.2)	0.75	**<0.0001**
BA (cm^2^)	2,025 ± 193^(*n*=27)^	2,041 ± 229	1,974 ± 250	1,840 ± 201^(*n*=30)^	1,820 ± 165	1,839 ± 241	1,781 ± 231^(*n*=29)^	1,703 ± 227^(*n*=30)^	−8.7 (−11.0, −6.5)	0.20	**<0.0001**	−−2.8 (−4.0, −1.7)	0.83	**<0.0001**
**Spine**														
aBMD (g/cm^2^)	1.1 ± 0.1^(*n*=27)^	1.0 ± 0.1	0.9 ± 0.2	0.8 ± 0.1^(*n*=30)^	0.8 ± 0.1	0.8 ± 0.2^(*n*=32)^	0.8 ± 0.1^(*n*=29)^	0.7 ± 0.1^(*n*=31)^	−0.008 (−0.01, −0.007)	0.34	**<0.0001**	−0.006 (−0.008, −0.005)	0.50	**<0.0001**
BMC (g)	53.3 ± 9.5^(*n*=27)^	48.7 ± 10.1	45.5 ± 10.1	37.6 ± 8.1^(*n*=30)^	38.2 ± 7.2	37.6 ± 10.2^(*n*=32)^	35.7 ± 9.2^(*n*=29)^	32.9 ± 8.5^(*n*=31)^	−0.5 (−0.6, −0.4)	0.29	**<0.0001**	−0.3 (−0.4, −0.2)	0.58	**<0.0001**
BA (cm^2^)	48.6 ± 3.6^(*n*=27)^	48.5 ± 4.9	48.5 ± 4.4	46.4 ± 5.4^(*n*=30)^	46.9 ± 4.3	47.7 ± 5.2^(*n*=32)^	45.6 ± 5.1^(*n*=29)^	44.9 ± 6.1^(*n*=31)^	−0.1 (−0.2, −0.1)	0.07	**<0.0001**	−0.007 (−0.05, 0.03)	0.47	0.734
**Total hip**														
aBMD (g/cm^2^)	1.0 ± 0.2^(*n*=27)^	1.0 ± 0.1^(*n*=30)^	0.9 ± 0.1	0.8 ± 0.1^(*n*=30)^	0.8 ± 0.1^(*n*=29)^	0.7 ± 0.1^(*n*=32)^	0.7 ± 0.1^(*n*=29)^	0.7 ± 0.1^(*n*=33)^	−0.009 (−0.01, −0.008)	0.44	**<0.0001**	−0.008 (−0.009, −0.006)	0.51	**<0.0001**
BMC (g)	28.6 ± 5.0^(*n*=27)^	28.1 ± 4.8^(*n*=30)^	25.0 ± 4.5	23.0 ± 3.3^(*n*=30)^	22.0 ± 3.2^(*n*=29)^	21.9 ± 3.6^(*n*=32)^	20.9 ± 4.4^(*n*=29)^	19.2 ± 3.9^(*n*=33)^	−0.2 (−0.3, −0.2)	0.35	**<0.0001**	−0.2 (−0.2, −0.1)	0.51	**<0.0001**
BA (cm^2^)	28.3 ± 2.1^(*n*=27)^	28.5 ± 1.9^(*n*=30)^	28.8 ± 2.1	28.3 ± 2.0^(*n*=30)^	28.5 ± 1.9^(*n*=29)^	29.6 ± 2.3^(*n*=32)^	28.4 ± 2.3^(*n*=29)^	28.6 ± 2.3^(*n*=33)^	0.004 (−0.02, 0.03)	0.0005	0.719	0.04 (0.02, 0.06)	0.39	**<0.0001**
**Femoral neck**														
aBMD (g/cm^2^)	1.0 ± 0.1^(*n*=27)^	1.0 ± 0.1^(*n*=30)^	0.9 ± 0.1	0.8 ± 0.1^(*n*=30)^	0.8 ± 0.1^(*n*=29)^	0.7 ± 0.1^(*n*=32)^	0.7 ± 0.1^(*n*=29)^	0.7 ± 0.1^(*n*=32)^	−0.008 (−0.01, −0.007)	0.45	**<0.0001**	−0.007 (−0.008, −0.006)	0.54	**<0.0001**
BMC (g)	4.1 ± 0.8^(*n*=27)^	4.0 ± 0.8^(*n*=30)^	3.6 ± 0.8	3.3 ± 0.7^(*n*=30)^	3.2 ± 0.6^(*n*=29)^	3.1 ± 0.7^(*n*=32)^	3.0 ± 0.7^(*n*=29)^	2.6 ± 0.7^(*n*=32)^	−0.04 (−0.04, −0.03)	0.29	**<0.0001**	−0.03 (−0.03, −0.02)	0.44	**<0.0001**
BA (cm^2^)	4.2 ± 0.7^(*n*=27)^	4.1 ± 0.6^(*n*=30)^	4.2 ± 0.6	4.1 ± 0.7^(*n*=30)^	4.1 ± 0.6^(*n*=29)^	4.2 ± 0.7^(*n*=32)^	4.2 ± 0.6^(*n*=29)^	3.9 ± 0.7^(*n*=32)^	−0.005 (−0.01, 0.002)	0.01	0.124	0.001 (−0.006, 0.008)	0.14	0.775
**Radius**														
aBMD (g/cm^2^)	0.6 ± 0.1^(*n*=27)^	0.6 ± 0.1	0.6 ± 0.1	0.5 ± 0.1^(*n*=29)^	0.5 ± 0.1	0.4 ± 0.1	0.5 ± 0.1	0.4 ± 0.1^(*n*=32)^	−0.006 (−0.007, −0.005)	0.44	**<0.0001**	−0.005 (−0.005, −0.004)	0.58	**<0.0001**
BMC (g)	9.0 ± 1.5^(*n*=27)^	8.8 ± 1.2	8.3 ± 1.8	7.0 ± 1.2^(*n*=29)^	7.1 ± 1.3	6.8 ± 1.6	6.6 ± 1.4	6.1 ± 1.2^(*n*=32)^	−0.08 (−0.09, −0.06)	0.31	**<0.0001**	−0.05 (−0.06, −0.04)	0.60	**<0.0001**
BA (cm^2^)	14.5 ± 1.3^(*n*=27)^	15.0 ± 1.7	14.7 ± 1.8	14.8 ± 1.5^(*n*=29)^	15.0 ± 1.6	15.6 ± 1.2	14.7 ± 1.5	15.3 ± 1.9^(*n*=32)^	0.01 (−0.003, 0.03)	0.01	0.103	0.04 (0.03, 0.05)	0.41	**<0.0001**

*Values are mean ± SD*.

*Bold indicates significance*.

*Adjustments were made for weight and height*.

*β-Coefficients are calculated with age as a continuous variable*.

*Superscript values indicate the group numbers*.

*aBMD, areal bone mineral density; BMC, bone mineral content; BA, bone area*.

**Table 7:** Peripheral quantitative computed tomography bone parameters in men.

**Age (yr)**	**40–44** (*n* = 25)	**45–49** (*n* = 34)	**50–54** (*n* = 29)	**55–59** (*n* = 31)	**60–64** (*n* = 27)	**65–69** (*n* = 35)	**70–74** (*n* = 30)	**75+** (*n* = 28)	**β-Coefficient (95% CI)**	***R*^2^**	**Unadjusted *p*-value**	**β-Coefficient (95% CI)**	***R*^2^**	**Adjusted *p*-value**

**Radius**														
4% Total vBMD (mg/cm^3^)	362.5 ± 45.1^(*n*=22)^	327.3 ± 39.6^(*n*=27)^	327.0 ± 45.0^(*n*=25)^	331.9 ± 56.2^(*n*=26)^	296.2 ± 52.5^(*n*=24)^	292.6 ± 55.8^(*n*=25)^	276.0 ± 46.2^(*n*=25)^	292.2 ± 57.5^(*n*=23)^	−1.92 (−2.5, −1.3)	0.18	**<0.0001**	−1.77 (−2.4, −1.2)	0.20	**<0.0001**
4% Trab.vBMD (mg/cm^3^)	206.5 ± 44.7^(*n*=22)^	181.6 ± 39.7^(*n*=27)^	173.1 ± 31.3^(*n*=25)^	177.3 ± 41.8^(*n*=26)^	152.3 ± 40.2^(*n*=24)^	147.9 ± 38.6^(*n*=25)^	149.6 ± 34.6^(*n*=25)^	140.2 ± 35.2^(*n*=23)^	−1.57 (−2.0, −1.1)	0.21	**<0.0001**	−1.45 (−1.9, −1.0)	0.23	**<0.0001**
33% Ct.vBMD (mg/cm^3^)	1,228.9 ± 35.8^(*n*=24)^	1,230.8 ± 37.8^(*n*=29)^	1,235.0 ± 33.8^(*n*=25)^	1,224.0 ± 34.1^(*n*=28)^	1,203.2 ± 44.9^(*n*=23)^	1,203.8 ± 25.9^(*n*=26)^	1,192.5 ± 30.1^(*n*=25)^	1,207.0 ± 31.4^(*n*=23)^	−0.96 (−1.4, −0.6)	0.10	**<0.0001**	−0.94 (−1.4, −0.5)	0.11	**<0.0001**
33% CSA (mm^2^)	139.5 ± 18.0^(*n*=24)^	135.2 ± 20.2^(*n*=29)^	142.8 ± 20.0^(*n*=25)^	140.9 ± 16.5^(*n*=28)^	142.6 ± 17.4^(*n*=23)^	134.7 ± 19.3^(*n*=26)^	138.8 ± 20.4^(*n*=25)^	133.4 ± 14.9^(*n*=23)^	−0.12 (−0.3, 0.1)	0.01	0.274	0.14 (−0.1, 0.3)	0.19	0.188
33% Ct.Th (mm)	2.7 ± 0.3^(*n*=24)^	2.7 ± 0.3^(*n*=29)^	2.8 ± 0.3^(*n*=25)^	2.7 ± 0.3^(*n*=28)^	2.6 ± 0.3^(*n*=23)^	2.6 ± 0.4^(*n*=26)^	2.4 ± 0.4^(*n*=25)^	2.5 ± 0.3^(*n*=23)^	−0.01 (−0.01, −0.01)	0.11	**<0.0001**	−0.01 (−0.01, −0.003)	0.16	**0.001**
33% SSI (mm^3^)	310.1 ± 0.3^(*n*=24)^	293.5 ± 63.0^(*n*=29)^	354.2 ± 80.3^(*n*=25)^	305.3 ± 69.8^(*n*=28)^	332.6 ± 61.7^(*n*=23)^	297.9 ± 84.6^(*n*=26)^	309.2 ± 86.0^(*n*=25)^	287.9 ± 51.5^(*n*=23)^	−0.60 (−1.4, 0.2)	0.01	0.150	0.32 (−0.5, 1.1)	0.17	**<0.0001**
**Tibia**														
4% Total vBMD (mg/cm^3^)	316.9 ± 43.7	283.0 ± 38.3^(*n*=30)^	285.2 ± 39.2^(*n*=25)^	275.8 ± 37.1^(*n*=28)^	255.1 ± 42.5^(*n*=25)^	259.8 ± 48.3^(*n*=32)^	245.5 ± 33.0^(*n*=27)^	249.9 ± 40.1^(*n*=24)^	−1.62 (−2.1, −1.2)	0.19	**<0.0001**	−1.45 (−1.9, −1.0)	0.31	**<0.0001**
4% Trab.vBMD (mg/cm^3^)	204.4 ± 40.3^(*n*=24)^	186.8 ± 33.9^(*n*=30)^	181.6 ± 29.3^(*n*=25)^	172.8 ± 26.9^(*n*=28)^	169.5 ± 32.2^(*n*=25)^	170.0 ± 37.0^(*n*=32)^	161.2 ± 25.6^(*n*=27)^	163.0 ± 30.0^(*n*=24)^	−0.96 (−1.3, −0.6)	0.12	**<0.0001**	−0.81 (−1.2, −0.5)	0.25	**<0.0001**
38% Ct.vBMD (mg/cm^3^)	1,219.8 ± 33.8	1,218.6 ± 29.0^(*n*=31)^	1,220.1 ± 37.1^(*n*=25)^	1,218.7 ± 39.6^(*n*=28)^	1,199.5 ± 42.6^(*n*=26)^	1,204.3 ± 31.0^(*n*=32)^	1,193.6 ± 35.5^(*n*=27)^	1,202.4 ± 32.1^(*n*=24)^	−0.62 (−1.0, −0.2)	0.05	**0.002**	−0.79 (−1.2, −0.4)	0.08	**<0.0001**
38% CSA (mm^2^)	472.2 ± 61.4	461.5 ± 64.3^(*n*=31)^	453.3 ± 52.2^(*n*=25)^	455.4 ± 39.1^(*n*=28)^	462.9 ± 66.6^(*n*=26)^	442.3 ± 55.1^(*n*=32)^	452.5 ± 63.4^(*n*=27)^	425.4 ± 38.8^(*n*=24)^	−0.87 (−1.5, −0.3)	0.04	**0.005**	0.004 (−0.6, 0.6)	0.28	0.989
38% Ct.Th (mm)	5.0 ± 0.5	5.0 ± 0.5^(*n*=31)^	5.0 ± 0.4^(*n*=25)^	4.9 ± 0.5^(*n*=28)^	4.6 ± 0.6^(*n*=26)^	4.7 ± 0.6^(*n*=32)^	4.8 ± 0.7^(*n*=27)^	4.8 ± 0.6^(*n*=24)^	−0.01 (−0.01, −0.001)	0.02	**0.026**	−0.004 (−0.01, 0.002)	0.07	0.203
38% SSI (mm^3^)	2,129.2 ± 360.9	2,043.5 ± 364.8^(*n*=31)^	2,033.3 ± 320.1^(*n*=25)^	2,046.3 ± 253.2^(*n*=28)^	1,928.8 ± 379.0^(*n*=26)^	1,896.7 ± 314.8^(*n*=32)^	1,949.7 ± 354.9^(*n*=27)^	1,839.5 ± 212.5^(*n*=24)^	−6.37 (−9.9, −2.9)	0.06	**<0.0001**	−1.54 (−4.9, 1.8)	0.27	0.362

*Values are mean ± SD. Bold indicates significance. Adjustments were made for weight and height. β-Coefficients are calculated with age as a continuous variable. Superscript values indicate the group numbers. vBMD, volumetric bone mineral density; Trab, trabecular; Ct, cortical; Th, thickness; CSA, cross-sectional area; SSI, stress strain index*.

**Table 8:** Peripheral quantitative computed tomography bone parameters in women.

**Age (yr)**	**40–44** (*n* = 28)	**45–49** (*n* = 32)	**50–54** (*n* = 30)	**55–59** (*n* = 31)	**60–64** (*n* = 31)	**65–69** (*n* = 33)	**70–74** (*n* = 30)	**75+** (*n* = 34)	**β-Coefficient (95% CI)**	***R*^2^**	**Unadjusted *p*-value**	**β-Coefficient (95% CI)**	***R*^2^**	**Adjusted *p*-value**

**Radius**														
4% Total vBMD (mg/cm^3^)	305.9 ± 46.1^(*n*=25)^	294.0 ± 47.3^(*n*=30)^	267.7 ± 45.5^(*n*=26)^	245.0 ± 34.9^(*n*=25)^	244.9 ± 43.6^(*n*=25)^	214.7 ± 42.8^(*n*=29)^	236.0 ± 39.1^(*n*=22)^	208.1 ± 39.1^(*n*=30)^	−2.57 (−3.0, −2.1)	0.36	**<0.0001**	−2.31 (−2.8, −1.8)	0.41	**<0.0001**
4% Trab.vBMD (mg/cm^3^)	146.5 ± 37.0^(*n*=25)^	144.5 ± 33.8^(*n*=30)^	122.3 ± 30.3^(*n*=26)^	114.4 ± 27.9^(*n*=25)^	106.5 ± 21.0^(*n*=25)^	97.8 ± 30.8^(*n*=29)^	104.0 ± 16.8^(*n*=22)^	83.9 ± 29.9^(*n*=30)^	−1.63 (−1.9, −1.3)	0.33	**<0.0001**	−1.52 (−1.9, −1.2)	0.35	**<0.0001**
33% Ct.vBMD (mg/cm^3^)	1,252.2 ± 39.8^(*n*=26)^	1,235.8 ± 46.4^(*n*=29)^	1,208.3 ± 52.8^(*n*=28)^	1,169.4 ± 46.6^(*n*=27)^	1,150.3 ± 35.7^(*n*=25)^	1,142.9 ± 52.5^(*n*=28)^	1,154.0 ± 37.5^(*n*=22)^	1,131.5 ± 45.5^(*n*=25)^	−3.28 (−3.8, −2.7)	0.42	**<0.0001**	−3.03 (−3.6, −2.5)	0.45	**<0.0001**
33% CSA (mm^2^)	104.8 ± 11.9^(*n*=26)^	109.0 ± 11.7^(*n*=29)^	108.8 ± 16.6^(*n*=28)^	108.9 ± 17.1^(*n*=27)^	109.6 ± 11.7^(*n*=25)^	111.0 ± 11.7^(*n*=28)^	105.5 ± 13.0^(*n*=22)^	115.6 ± 16.7^(*n*=25)^	0.18 (0.02, 0.3)	0.02	**0.027**	0.33 (0.2, 0.5)	0.23	**<0.0001**
33% Ct.Th (mm)	2.4 ± 0.3^(*n*=26)^	2.3 ± 0.3^(*n*=29)^	2.2 ± 0.4^(*n*=28)^	1.8 ± 0.3^(*n*=27)^	1.8 ± 0.3^(*n*=25)^	1.7 ± 0.4^(*n*=28)^	1.7 ± 0.4^(*n*=22)^	1.5 ± 0.3^(*n*=25)^	−0.02 (−0.03, −0.02)	0.43	**<0.0001**	−0.02 (−0.02, −0.02)	0.50	**<0.0001**
33% SSI (mm^3^)	215.5 ± 37.4^(*n*=26)^	222.7 ± 34.8^(*n*=29)^	211.6 ± 53.3^(*n*=28)^	189.0 ± 37.1^(*n*=27)^	194.4 ± 34.5^(*n*=25)^	193.7 ± 126.2^(*n*=28)^	185.8 ± 33.3^(*n*=22)^	191.0 ± 43.1^(*n*=25)^	−0.89 (−1.3, −0.4)	0.07	**<0.0001**	−0.39 (−0.8, 0.01)	0.29	0.06
**Tibia**														
4% Total vBMD (mg/cm^3^)	290.1 ± 36.0^(*n*=26)^	276.5 ± 38.9^(*n*=30)^	255.5 ± 36.8^(*n*=30)^	225.5 ± 29.4^(*n*=28)^	224.2 ± 30.4^(*n*=27)^	201.1 ± 35.5^(*n*=31)^	206.6 ± 38.2^(*n*=25)^	187.2 ± 36.7^(*n*=31)^	−2.72 (−3.1, −2.3)	0.47	**<0.0001**	−2.5 (−2.9, −2.1)	0.55	**<0.0001**
4% Trab.vBMD (mg/cm^3^)	195.5 ± 28.6^(*n*=26)^	186.3 ± 33.6^(*n*=30)^	163.9 ± 32.0^(*n*=30)^	141.0 ± 32.1^(*n*=28)^	139.5 ± 25.5^(*n*=27)^	133.8 ± 34.0^(*n*=31)^	137.6 ± 30.9^(*n*=25)^	119.0 ± 31.6^(*n*=31)^	−1.90 (−2.2, −1.57)	0.35	**<0.0001**	−1.65 (−2.0, −1.3)	0.43	**<0.0001**
38% Ct.vBMD (mg/cm^3^)	1,234.9 ± 43.4^(*n*=26)^	1,215.1 ± 49.9^(*n*=30)^	1,197.3 ± 46.2^(*n*=30)^	1,161.8 ± 44.8^(*n*=28)^	1,145.0 ± 49.0^(*n*=27)^	1,144.4 ± 53.8^(*n*=31)^	1,147.7 ± 47.0^(*n*=25)^	1,128.2 ± 55.3^(*n*=31)^	−2.64 (−3.2, −2.1)	0.29	**<0.0001**	−2.45 (−3.0, −1.9)	0.31	**<0.0001**
38% CSA (mm^2^)	362.0 ± 49.7^(*n*=26)^	363.2 ± 43.1^(*n*=30)^	355.5 ± 48.5^(*n*=30)^	363.8 ± 43.5^(*n*=28)^	359.1 ± 35.7^(*n*=27)^	373.8 ± 56.5^(*n*=31)^	334.5 ± 46.4^(*n*=25)^	360.4 ± 52.7^(*n*=31)^	−0.16 (−0.7, 0.3)	0.01	0.530	0.48 (−0.001, 1.0)	0.22	0.050
38% Ct.Th (mm)	4.1 ± 0.6^(*n*=26)^	4.0 ± 0.6^(*n*=30)^	3.9 ± 0.4^(*n*=30)^	3.7 ± 0.5^(*n*=28)^	3.5 ± 0.6^(*n*=27)^	3.6 ± 0.7^(*n*=31)^	3.3 ± 0.7^(*n*=25)^	3.0 ± 0.6^(*n*=31)^	−0.03 (−0.03, −0.02)	0.23	**<0.0001**	−0.02 (−0.03, −0.01)	0.33	**<0.0001**
38% SSI (mm^3^)	1,430.8 ± 271.2^(*n*=26)^	1,359.2 ± 226.1^(*n*=30)^	1,353.3 ± 283.8^(*n*=30)^	1,350.1 ± 224.8^(*n*=28)^	1,244.8 ± 146.2^(*n*=27)^	1,338.9 ± 313.4^(*n*=31)^	1,134.5 ± 250.5^(*n*=25)^	1,175.1 ± 227.5^(*n*=31)^	−6.34 (−9.0, −3.7)	0.09	**<0.0001**	−2.55 (−5.0, −0.1)	0.34	**0.038**

*Values are mean ± SD*.

*Bold indicates significance*.

*Adjustments were made for weight and height*.

*β-Coefficients are calculated with age as a continuous variable*.

*Superscript values indicate the group numbers*.

*vBMD, volumetric bone mineral density; Trab, trabecular; Ct, cortical; Th, thickness; CSA, cross-sectional area; SSI, stress strain index*.

From the LVA scans, 9% of GamBAS participants had moderate or severe vertebral fractures (as defined by GE Lunar software), and 14% had spinal degeneration (osteophytes present). Hip fractures were self-reported; 3% of women and 0.4% of men reported a hip fracture, or fracture-like injury. Comparing the GamBAS participants to the manufacturers US Black reference database showed the population to have lower age, and gender-matched *Z*-scores for aBMD than the reference for both the lumbar spine and hip. The mean (SD), range of those *Z*-scores are: lumbar spine L1–L4, women −2.0 (1.1), −4.7 to 1.5; men −1.5 (1.3), −4.4 to 3.9 and for femoral neck, women −1.1 (0.9), −3.3 to 2.0; men −1.0 (0.9), −3.2 to 1.7. Peripheral vascular calcification in the lower-limb was visible in 16% of the population. There were no sex differences in the presence of peripheral vascular calcification: 19% in men vs 15% in women, *p* = 0.195.

## Discussion

GamBAS is the first and largest prospective longitudinal study in West Africa in which quantitative measurements of bone and muscle have been collected. The initial findings have highlighted that women have a greater degree of aBMD loss compared to men, yet have less loss of muscle. Analyses of longitudinal data will allow more accurate quantification of this loss and investigation of the mechanisms driving these effects. Data from DXA demonstrate that height-adjusted BA increased with age at the total hip and radius in both men and women; however, pQCT revealed that the increase in cross-sectional BA at the radius was only evident in women. The decrease in cortical thickness in women suggests this increase in cross-sectional BA may be a biomechanical adaptation to loss of bone. However, whether this is indicative of a cohort effect or age-related periosteal apposition will be confirmed in analysis of prospective data. In agreement with our previous work, the magnitude of age-differences is similar to that observed in other populations, where fracture incidence is higher.

Our preliminary analyses of cross-sectional data show that total hip aBMD was negatively associated with age in Gambian men and women. Self-reported hip fracture rates were 0.4–3% and lower compared to sex- and age-adjusted hip fracture incidence elsewhere in the world (32). The aBMD *Z*-scores we report show that Gambian men and women had osteoporosis at the lumbar spine compared to the manufacturer’s reference US Black population. However, this population may not be the most appropriate one to use as a reference and may have led to overestimating the prevalence of osteoporosis in the population (33, 34). Using the LVA scans, for the first time we report the prevalence of vertebral fractures in this cohort as 9%, with 6% in men and 3% in women. The prevalence of vertebral fractures among GamBAS participants is consistent with a recent report in South African women, where prevalence was 9 and 5% in black and white women, respectively (35). The positive association between spine BA and age in men may be due to spinal degeneration artefact and requires further investigation.

Calcium is one of the main mineral components of the skeletal system, and adequate dietary intake is essential for healthy bones. In adults from high-income countries, low calcium intake has been associated with an increased risk of osteoporosis, fractures, and falls (36). We report that habitual dietary calcium intake across the age bands in Gambian adults was low relative to international recommendations, for example in the UK the reference nutrient intake for adults is 700 mg/day (37) and in the US and Canada 1,000–1,200 mg/d (38). Furthermore, dietary calcium intake was low in Gambians compared to UK adults (743–912 mg/day) (39) and American adults (748–1,209 mg/day) (40). Future analyses of longitudinal data investigating the relationship between dietary intake and musculoskeletal health in Gambian men and women are therefore warranted. It is important to note that in our groups other work in children and women of reproductive age, increasing calcium intake did not have a lasting benefit, and findings contradict those that we would expect in countries where habitual intakes are much higher (18, 41–45).

The strengths and potential limitations of GamBAS merit consideration. This was the first study of ageing in The Gambia and we did not know how well the study would be accepted by the older community in Kiang West. We achieved our sample target within each of our age-stratified groups; the stratified study design ensured equal distribution of sampling across the age bands of men and women recruited. Our suite of measurements is unique and allows detailed characterisation of musculoskeletal outcomes. The long-standing relationship between the population of Kiang West and MRC Keneba is maintained through high levels of communication and interaction with a “research-friendly” population. The KWDSS information dates back to the 1950’s so that accurate dates of birth for determination of age in the older-age bands is possible and also facilitated study design and recruitment stratification by age and village.

We could not accurately assess menopausal status from the women’s health questionnaire as some questions did not translate well in the local language leading to errors in information. Fertility status is sensitive in The Gambian culture and so future studies investigating how best to determine menopausal status are required. It was not possible to distinguish secular differences in attained adult size (older men and women were born at a time when early growth faltering may have been more severe) from age-related changes from the reported cross-sectional data. However, this will be possible with the longitudinal data. The majority of recruits were from the four core villages which may be healthier than the other villages as they are located closer to MRC Keneba where the residents are able to receive medical care. This, and that we only needed to recruit 30% of the Kiang West residents, to achieve our target sample size, may cause bias in our findings. In The Gambia, traditional bone-setters tend to be used for the treatment of musculoskeletal disorders, including fractures, and the information is not added to clinic medical records. Therefore, the hip fracture data obtained are based on self-report and may be inaccurate or incomplete because verification through radiograph reports is not possible. The vertebral fracture data are from GE Lunar Prodigy scans; in later follow-up using the higher-resolution iDXA, we will be able to more accurately assess vertebral fractures. There was difficulty in obtaining medical records from the participants who were not living in the four core villages of Kiang West. This is a homogenous population with little variance in lifestyle characteristics. When comparing to other regions, sub-Saharan Africa or the globe, it will be important to investigate further the effects of environment such as physical activity levels and occupation. Finally, Kiang West is one region of The Gambia and the data may not be generalizable to urban areas of the country, to other West African countries or to the remainder of the continent.

In conclusion, the GamBAS cohort provides important information on the aetiology of ageing in a rural sub-Saharan setting. This is crucial as the ageing population increases and with it, NCD burden.

## Collaboration

The MRC has a long and successful history of conducting collaborative research and we welcome specific proposals for new collaborations. Data sharing is available through collaborative agreements and initial enquiries should be made to the Principal Investigator Kate Ward (kw@mrc.soton.ac.uk).

## Ethics Statement

Ethics was obtained from the MRC Unit The Gambia Scientific Co-ordinating Committee (SCC) and joint Gambian Government/MRC Unit The Gambia Ethics committee (SCC#1222). All participants provided written informed consent. All procedures were carried out in accordance with the Declaration of Helsinki (28). If any participant had potential health problems identified by fieldworkers during recruitment or by the team during the field visit, they were advised to visit the clinic at MRC Keneba for follow-up and were offered transport. Participants with elevated blood pressure [according to WHO guidelines (29)] were followed up and treated as appropriate. Any other abnormalities were discussed with the study physician who followed-up appropriately. For instance, if musculoskeletal abnormalities were detected on the scan, the participant was referred for radiography at MRC Fajara.

## Author Contributions

KW, AP, GG, IS, AF, LJ, and YS designed the study; KW, AF, IS, GG, LJ, and YS contributed to the data collection; AZ and KW analysed the data from the study; AZ, KW, AP, IS, and GG interpreted the data from the study; AZ, KW, AF, and AP drafted the manuscript; AZ, KW, AP, GG, IS, AF, LJ, and YS revised the manuscript; and KW gave the final approval of the version to be published.

## Conflict of Interest Statement

The authors declare that the research was conducted in the absence of any commercial or financial relationships that could be construed as a potential conflict of interest.
