# Corrigendum: The Gambian Bone and Muscle Ageing Study: Baseline Data From a Prospective Observational African Sub-Saharan Study

**DOI:** 10.3389/fendo.2018.00160

**Published:** 2018-04-16

**Authors:** Ayse Zengin, Anthony J. Fulford, Yankuba Sawo, Landing M. Jarjou, Inez Schoenmakers, Gail Goldberg, Ann Prentice, Kate A. Ward

**Affiliations:** ^1^Nutrition and Bone Health Group, MRC Elsie Widdowson Laboratory, Cambridge, United Kingdom; ^2^Faculty of Medicine, Nursing and Health Sciences, Department of Medicine, School of Clinical Sciences at Monash Health, Monash University, Monash Medical Centre, Clayton, VIC, Australia; ^3^International Nutritional Group, London School of Hygiene and Tropical Medicine, London, United Kingdom; ^4^Calcium, Vitamin D and Bone Health Group at MRC Unit The Gambia, Banjul, Gambia; ^5^Faculty of Medicine and Health Sciences, Department of Medicine, University of East Anglia, Norwich, United Kingdom; ^6^MRC Lifecourse Epidemiology Unit, University of Southampton, Southampton, United Kingdom

**Keywords:** bone, ageing, Africa, muscle, dual energy X-ray absorptiometry, non-communicable disease, Gambia

There was a mistake in the values in Table 4 in the parameters total % fat, android fat mass, gynoid fat mass, aLM, android lean mass, and gynoid lean mass. The correct version of Table 4 appears below. The authors apologize for the mistake. This error does not change the scientific conclusions of the article.

The nutritional intake data in Table 2 was incorrectly labeled. The correct version of Table 2 appears below. We have also edited the interpretation of the data in the Results section from:

Overall, women had higher intakes of all micronutrients. Some notable sex differences include a 21% greater daily habitual calcium intake in women than in men (Table 2). The greatest sex difference was seen in daily habitual iron intake, where women had a 33% greater daily iron intake compared to men. Across the age bands, daily habitual calcium intake [mean (SD)] was 295.9 (175.9) mg/day in men and 378.0 (176.0) mg/day in women (Table 2).

**Table 2:** Nutritional intake of men and women.

	Men (*n* = 225)^a^	Women (*n* = 242)^a^	*p*-value
Calcium (mg/day)	378.0 ± 176.0	295.9 ± 175.9	**<0.0001**
Phosphorus (mg/day)	836.4 ± 275.4	620.2 ± 243.4	**<0.0001**
Iron (mg/day)	37.2 ± 25.8	25.0 ± 16.5	**<0.0001**
Zinc (mg/day)	9.3 ± 3.0	7.0 ± 2.8	**<0.0001**
Dietary fibres (mg/day)	44.4 ± 14.2	33.9 ± 12.4	**<0.0001**
Phytate (g/day)	1.3 ± 0.5	1.0 ± 0.4	**<0.0001**
Potassium (mg/day)	2,409.0 ± 868.9	1,800.1 ± 705.4	**<0.0001**
Magnesium (mg/day)	527.3 ± 192.9	388.4 ± 150.4	**<0.0001**

*Values are mean ± SD*.

*Bold indicates significance*.

*Dietary intakes were estimated from 2-day weighed diet diaries, and intakes calculated from Gambian food tables*.

*^a^21 participants did not have dietary information available*.

**Table 4:** Anthropometry and body composition in women.

	40–44 (*n* = 28)	45–49 (*n* = 32)	50–54 (*n* = 30)	55–59 (*n* = 31)	60–64 (*n* = 31)	65–69 (*n* = 33)	70–74 (*n* = 30)	75+ (*n* = 34)	β-coefficient (95% CI)	*p*-value
Weight (kg)	58.1 ± 11.5	60.8 ± 11.4	57.1 ± 10.8	53.8 ± 9.6	53.4 ± 7.2	53.5 ± 9.6	52.2 ± 9.9	49.3 ± 8.5	−0.26 (−0.35, −0.16)	**<0.0001**
Height (cm)	159.3 ± 5.1	159.8 ± 6.1	158.6 ± 6.2	158.1 ± 5.8	157.6 ± 4.9	160.1 ± 5.7	154.8 ± 5.7	154.0 ± 5.7	−0.14 (−0.20, −0.09)	**<0.0001**
Sitting height (cm)	81.7 ± 2.8	81.2 ± 3.5	80.4 ± 2.9	79.1 ± 3.8	79.5 ± 3.1	80.2 ± 3.5	77.8 ± 3.3	76.5 ± 3.3	−0.13 (−0.16, −0.09)	**<0.0001**
Sit:Stand height ratio	0.51 ± 0.02	0.51 ± 0.01	0.51 ± 0.02	0.50 ± 0.02	0.50 ± 0.01	0.50 ± 0.02	0.50 ± 0.02	0.50 ± 0.01	−0.0004 (−0.0005, −0.0002)	**<0.0001**
BMI	22.9 ± 4.4	23.9 ± 4.4	22.7 ± 4.3	21.4 ± 3.1	21.4 ± 2.3	20.8 ± 3.2	21.7 ± 3.7	20.7 ± 2.8	−0.07 (−0.10, −0.03)	**<0.0001**
Waist circumference (cm)	70.7 ± 10.1	75.7 ± 9.7	72.0 ± 8.6	70.6 ± 6.6	71.4 ± 6.3^(^*^n^*^=29)^	71.0 ± 7.1^(^*^n^*^=29)^	73.3 ± 8.5^(^*^n^*^=23)^	68.4 ± 5.4^(^*^n^*^=19)^	−0.06 (−0.14, 0.03)	0.203
Total body fat mass (kg)	18.4 ± 8.7^(^*^n^*^=27)^	20.7 ± 9.3	18.3 ± 8.3	16.3 ± 6.7^(^*^n^*^=30)^	16.0 ± 4.8	16.1 ± 6.8	16.4 ± 6.7^(^*^n^*^=29)^	14.1 ± 5.5^(^*^n^*^=30)^	−0.12 (−0.20, −0.05)	**0.001**
Total % fat	30.3 ± 8.1^(^*^n^*^=27)^	32.5 ± 10.0	30.8 ± 8.7	29.3 ± 8.2^(^*^n^*^=30)^	29.6 ± 6.0	29.0 ± 7.6	30.4 ± 7.9^(^*^n^*^=29)^	27.9 ± 7.1^(^*^n^*^=30)^	−0.07 (−0.15, 0.01)	0.09
Android fat mass (kg)	1.1 ± 0.9^(^*^n^*^=27)^	1.3 ± 0.8	1.2 ± 0.8	0.9 ± 0.5^(^*^n^*^=30)^	0.9 ± 0.4	1.0 ± 0.6	1.0 ± 0.6^(^*^n^*^=29)^	0.8 ± 0.5^(^*^n^*^=31)^	−0.008 (−0.01, −0.001)	**0.02**
Gynoid fat mass (kg)	4.1 ± 1.5^(^*^n^*^=27)^	4.3 ± 1.6	3.9 ± 1.3	3.4 ± 1.2	3.5 ± 1.0	3.4 ± 1.2	3.2 ± 1.1	2.9 ± 1.0^(^*^n^*^=33)^	−0.03 (−0.04, −0.02)	**<0.0001**
FMI (kg/m^2^)	7.2 ± 3.4^(^*^n^*^=27)^	8.1 ± 3.7	7.3 ± 3.4	6.5 ± 2.6^(^*^n^*^=30)^	6.4 ± 1.8	6.2 ± 2.5	6.9 ± 2.7^(^*^n^*^=29)^	6.0 ± 2.2^(^*^n^*^=30)^	−0.04 (−0.07, −0.01)	**0.009**
Total body lean mass (kg)	36.7 ± 4.1^(^*^n^*^=27)^	37.0 ± 4.4	35.7 ± 4.0	35.0 ± 4.5^(^*^n^*^=30)^	34.7 ± 3.6	34.7 ± 3.4	33.4 ± 4.7^(^*^n^*^=29)^	32.5 ± 4.3^(^*^n^*^=30)^	−0.11 (−0.16, −0.07)	**<0.0001**
aLM (kg)	16.9 ± 2.3^(^*^n^*^=27)^	16.9 ± 2.2	16.1 ± 2.3	15.6 ± 2.3	15.4 ± 2.1	15.3 ± 2.0	14.7 ± 2.4	14.1 ± 2.2	−0.07 (−0.10, −0.05)	**<0.0001**
Android lean mass (kg)	2.3 ± 0.3^(^*^n^*^=27)^	2.4 ± 0.4	2.3 ± 0.3	2.2 ± 0.3^(^*^n^*^=30)^	2.2 ± 0.2	2.2 ± 0.2	2.2 ± 0.4^(^*^n^*^=29)^	2.2 ± 0.3^(^*^n^*^=31)^	−0.006 (−0.009, −0.003)	**0.001**
Gynoid lean mass (kg)	5.2 ± 0.9^(^*^n^*^=27)^	5.2 ± 0.7	5.0 ± 0.6	4.7 ± 0.8	4.8 ± 0.7	4.8 ± 0.7	4.5 ± 0.7	4.4 ± 0.6^(^*^n^*^=33)^	−0.02 (−0.03, −0.01)	**<0.0001**
aLMI (kg/m^2^)	6.6 ± 0.8^(^*^n^*^=27)^	6.6 ± 0.8	6.4 ± 0.7	6.2 ± 0.6	6.2 ± 0.7	5.9 ± 0.6	6.1 ± 0.8	5.9 ± 0.7	−0.02 (−0.03, −0.01)	**<0.0001**

*Values are mean ± SD*.

*β-coefficients are calculated with age as a continuous variable*.

*Superscript values indicate the group numbers*.

*Bold indicates significance*.

*BMI, body mass index; FMI, fat mass index, calculated as whole body fat mass divided by height squared; aLM, appendicular lean mass; aLMI, appendicular lean mass index, calculated as appendicular lean mass divided by height squared*.

To:

Overall, men had higher intakes of all micronutrients. Some notable sex differences include a 21% greater daily habitual calcium intake in men than in women (Table 2). The greatest sex difference was seen in daily habitual iron intake, where men had a 33% greater daily iron intake compared to women. Across the age bands, daily habitual calcium intake [mean (SD)] was 378.0 (176.0) mg/day in men and 295.9 (175.9) mg/day in women (Table 2).

This error does not change the scientific conclusions of the article in any way.

The original article has been updated.

## Conflict of Interest Statement

The authors declare that the research was conducted in the absence of any commercial or financial relationships that could be construed as a potential conflict of interest.

