# Supplementary material for: The Gambian Bone and Muscle Ageing Study: Baseline Data from a Prospective Observational African Sub-Saharan Study
Source: Front Endocrinol (Lausanne). 2017 Aug 31;8:219. doi: 10.3389/fendo.2017.00219 (PMC5583153; doi:10.3389/fendo.2017.00219)
Supplement: Supplementary file 1 [file Data_Sheet_1.docx]

Supplementary Material

The Gambian Bone and Muscle Ageing Study (GamBAS): baseline data from a prospective observational sub-Saharan study

Ayse Zengin^1,2^, Anthony J Fulford^3^, Yankuba Sawo^4^, Landing M Jarjou^4^, Inez Schoenmakers^1,5^, Gail Goldberg^1,4^, Ann Prentice^1,4#^, Kate A Ward^1,6#*^

*** Correspondence:** Dr Kate Ward [kw@mrc.soton.ac.uk](mailto:kw@mrc.soton.ac.uk)

# Supplementary Information: Design to improve estimation of seasonal patterns

The seasonal pattern of variation of a variable is estimated from a series of measurements across the year. There are two extreme approaches to achieving this:

1. Each measurement is taken from a different individual.

2. All measurements are taken from the same individual.

Often each individual is subject to the same pattern of the seasonality so the two approaches would estimate the same pattern. However, this is not necessarily so. If individuals differ in the intensity with which they respond to seasonal changes then the two approaches would yield the same pattern although its amplitude may differ. The two clearly would not yield the same pattern if individual responses were out of phase with one another or differed in more complex ways. Supposing individual patterns were essentially the same, the second approach would be more accurate since it would not be clouded by other between-individual differences.

In this study we planned to make two measurements on each of many individuals. This gives us the potential to utilise within-individual information and reduce error due to between-individual variability, especially if all individuals respond essentially identically to seasonal changes. (In theory several observations on each individual could allow us to examine between-individual differences in the pattern of seasonality. However, individual seasonal patterns may differ in a variety of ways, each requiring one or more parameter estimate, yet two observations per individual only allows estimation of a single parameter and even this would be hard to distinguish from observation-level noise.) If individuals do all show the same season patterns we nevertheless extract zero within-individual information about this pattern if all individuals are re-measured a whole number of years after their first measurement and the SEs of seasonality (Fourier) coefficients remain high. The same is true for at least some of the Fourier coefficients for all fixed follow-up intervals no matter how long that interval is. The only solution therefore is to vary the follow-up interval between individuals. Simulations show that, with sufficient between-individual variation, the SE of all Fourier coefficients can be dramatically improved if the follow-up intervals differ randomly between 1.5 and 2 cycles.
